# Cost-effectiveness of improvement strategies for reperfusion treatments in acute ischemic stroke: a systematic review

**DOI:** 10.1186/s12913-023-09310-0

**Published:** 2023-03-30

**Authors:** Chi Phuong Nguyen, Willemijn J. Maas, Durk-Jouke van der Zee, Maarten Uyttenboogaart, Erik Buskens, Maarten M. H. Lahr, on behalf of the CONTRAST consortium

**Affiliations:** 1 Department of Operations, Faculty of Economics and Business, University of Groningen, Groningen, the Netherlands; 2 Department of Epidemiology, University of Groningen, University Medical Center Groningen, Groningen, the Netherlands; 3 Department of Pharmaceutical Administration and Economics, Hanoi University of Pharmacy, Hanoi, Vietnam; 4 Department of Neurology, University of Groningen, University Medical Center Groningen, Groningen, the Netherlands; 5 Department of Radiology, Medical Imaging Center, University of Groningen, University Medical Center Groningen, Groningen, the Netherlands

**Keywords:** Stroke, Time delay, Cost-effectiveness, Systematic review

## Abstract

**Background:**

Reducing delays along the acute stroke pathway significantly improves clinical outcomes for acute ischemic stroke patients eligible for reperfusion treatments. The economic impact of different strategies reducing onset to treatment (OTT) is crucial information for stakeholders in acute stroke management. This systematic review aimed to provide an overview on the cost-effectiveness of several strategies to reduce OTT.

**Methods:**

A comprehensive literature search was conducted in EMBASE, PubMed, and Web of Science until January 2022. Studies were included if they reported 1/ stroke patients treated with intravenous thrombolysis and/or endovascular thrombectomy, 2/ full economic evaluation, and 3/ strategies to reduce OTT. The Consolidated Health Economic Evaluation Reporting Standards statement was applied to assess the reporting quality.

**Results:**

Twenty studies met the inclusion criteria, of which thirteen were based on cost-utility analysis with the incremental cost-effectiveness ratio per quality-adjusted life year gained as the primary outcome. Studies were performed in twelve countries focusing on four main strategies: educational interventions, organizational models, healthcare delivery infrastructure, and workflow improvements. Sixteen studies showed that the strategies concerning educational interventions, telemedicine between hospitals, mobile stroke units, and workflow improvements, were cost-effective in different settings. The healthcare perspective was predominantly used, and the most common types of models were decision trees, Markov models and simulation models. Overall, fourteen studies were rated as having high reporting quality (79%-94%).

**Conclusions:**

A wide range of strategies aimed at reducing OTT is cost-effective in acute stroke care treatment. Existing pathways and local characteristics need to be taken along in assessing proposed improvements.

**Supplementary Information:**

The online version contains supplementary material available at 10.1186/s12913-023-09310-0.

## Background

Worldwide, stroke is the second leading cause of death and the most common cause of permanent disability, resulting in huge societal and economic burdens related to long-term care, rehabilitation, and productivity loss [1, 2]. Acute ischemic stroke (AIS) represents the majority of stroke patients, and reperfusion treatments like intravenous thrombolysis (IVT) and endovascular thrombectomy (EVT) have shown to be effective in improving functional outcomes. Both treatments are highly time-dependent, with IVT effective up to 4.5 h after symptom onset and EVT within 6 h [3, 4]. For selected patients suspected of a large vessel occlusion (LVO), EVT has shown to be effective even up to 24 h [5]. Importantly, shorter time from onset to treatment (OTT) with IVT or EVT is associated with better functional outcomes [6–8].

Due to the time dependency of reperfusion treatments, multiple strategies or interventions have been proposed to reduce time delays along the acute stroke pathway. Examples include educational interventions to create awareness among citizens to contact emergency services immediately following symptom onset [9], a mobile stroke unit that brings IVT to the patient instead of transporting the patients to the nearest IVT capable facility [9], telemedicine solutions for expert opinion at a distance [10], and workflow improvements including inter-hospital patients transfer, teamwork and feedback on performance [9, 11]. In addition, direct transport of acute stroke patients suspected of LVO from the onset scene to a comprehensive stroke center based on prehospital triage instruments [12] is a promising alternative organizational model, which could decrease the OTT time for patients eligible for EVT [13].

While several strategies have been developed to reduce time to reperfusion treatments, less is known about their cost-effectiveness. As stroke incidence and its burden on society are expected to increase [14], evidence generated by economic evaluations of these strategies will support policymakers, clinicians, and other stakeholders in deciding how to allocate scarce resources whilst optimizing clinical outcomes for patients. Therefore, the aim of this study is to systematically review the cost-effectiveness of strategies directed at reducing time to reperfusion treatments for AIS patients.

## Methods

### Search strategy and study selection

This systematic review was performed according to the Preferred Reporting Items for Systematic Reviews and Meta-Analyses (PRISMA) guidelines [15]. The search strategy was constructed by the first two authors (C.P.N. and W.J.M). Three electronic databases (EMBASE, MEDLINE/ Pubmed, and Web of Science) were searched to identify relevant articles published between January 2010 to January 2022. The search strategy was based on the PICOS format: The population (P) were ‘stroke’ patients, the intervention (I) ‘EVT or IVT’ and ‘reducing time-to-treatment’, and the outcome (O) was ‘incremental cost-effectiveness ratio’ (ICER). Comparators (C) and study design (S) were not included to maximize records retrieved. Our eligibility criteria were: (1) stroke patients treated with IVT and/or EVT, (2) full economic evaluation was performed (i.e., cost-effectiveness, cost-utility, or cost–benefit analysis), and (3) strategies or interventions that aim to reduce time to treatment or increasing the proportion of eligible patients for treatment due to time delay reduction. Papers were excluded if: (1) articles published before 2010; (2) articles not written in English, and (3) reviews, protocol papers, conference abstracts, letters to editors, or case reports. All identified records from three databases were stored in Endnote® X8 software [16]. Details of the search strategy are reported in the Supplementary Information (Appendix 1). In addition, a manual cross-reference of selected papers was performed to include more relevant papers.

Study selection, data extraction, and quality assessment were performed independently by two reviewers (C.P.N. and W.J.M.). Disagreements were discussed between reviewers until a consensus was reached. Discrepancies were resolved by consulting a third reviewer (M.M.H.L.).

### Data extraction and analysis

The following information was extracted from the selected papers independently by two reviewers (C.P.N. and W.J.M): country of origin, strategies including intervention, comparator, type of economic evaluation, effectiveness measurement, model type (i.e. Markov model, decision tree or other), economic perspective, time horizon, discount rate, reference year of cost, outcomes including incremental costs, incremental effectiveness and an ICER. An economic evaluation was stratified by type of strategy or intervention and compared with the same effectiveness measurement. An ICER was calculated by dividing the different costs by the different effectiveness of two strategies. The strategy or intervention was considered cost-effective if an ICER was less than the willingness-to-pay threshold mentioned in the study. In case specific willingness-to-pay thresholds were not stated in the study, the recommended threshold of one Gross Domestic Product (GDP) per capita was used according to the World Health Organization for cost-effectiveness studies [17]. The strategy or intervention was dominant if the strategy resulted in higher effectiveness (i.e., QALY) and less costs than the comparator. In case data were insufficient or unclear for data analysis, additional information was requested by contacting the corresponding author of individual papers.

### Quality assessment

For quality assessment, the Consolidated Health Economic Evaluation Reporting Standards (CHEERS) guideline [18] was used, scoring a total of 24 criteria. Papers were classified into three categories: high (75.0% or more), medium (from 50.0% to 74.9%) and low quality (less than 50.0%) [19, 20].

## Results

### Study selection

**Fig. 1:**
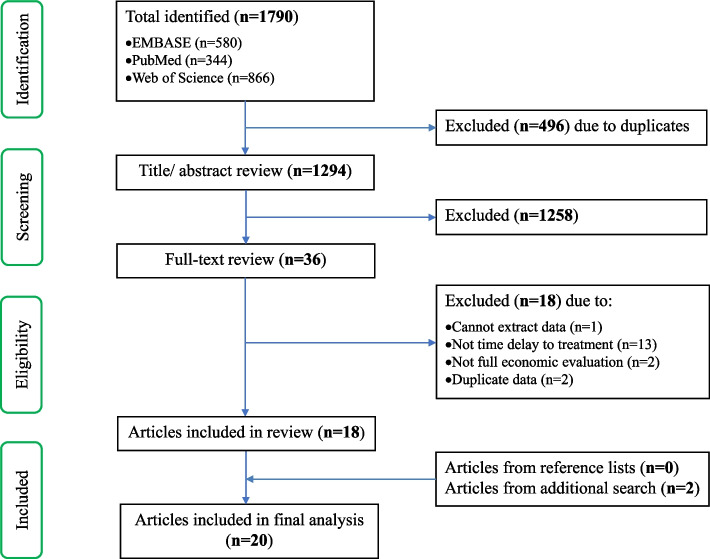
PRISMA flow chart

The initial literature search resulted in 1,790 records (Fig. 1). After removing duplicates, 1,294 articles were screened on title and abstract independently by both reviewers, and 36 articles were considered relevant for the full-text review. Of these articles, 18 articles were excluded because of missing outcome measures (*n* = 1), missing time to treatment variables (*n* = 13), missing full economic evaluation (*n* = 2) and data duplication between articles (*n* = 2). Two additional articles were identified when an additional search in Pubmed, EMBASE, and Web of Science was conducted until January 9^th^ 2022. Ultimately, 20 studies were included.

### Study characteristics

The majority of economic evaluations were conducted in high-income countries, and only one study was performed in an upper-middle-income country (China) [21]. Five studies were based in the United States [22–26], one study in Canada [27], 10 studies in Europe [28–37], one study in Japan [38], one study in Singapore [39], and one study in Australia [40]. The type of interventions varied across studies, including educational interventions [23, 34, 39], organizational models [22, 24–27, 30–32, 36, 38, 40], healthcare delivery infrastructure [29, 33, 35], and workflow improvement [28, 34]. An overview of all study characteristics is provided in Table 1.

**Table 1:** Study characteristics

Study	Country of origin	Strategy/ Intervention	Comparator	Type of economic evaluation	Outcome of effectiveness	Type of model	Economic perspective	Time horizon (Discount rate)	Reference year of cost
Ajmi (2021) [37]	Norway	Quality improvement project, including streamlining stroke care pathway^a^ and simulation-based training	No quality improvement project	Cost-effectiveness	Door-to-needle times, 90-day all-cause mortality	Economic evaluation along with clinical trial	Health care	5 years (NR)	2019
Tan (2021) [21]	China	Telemedicine between hub and spoke centers	No telemedicine	Cost-utility	QALY	Decision tree and Markov model	Societal and healthcare	30 years (3%)	2019
Coughlan (2021) [28]	England	Inter-hospital transfer by helicopter EMS	Ground EMS	Cost-utility	QALY	Decision tree and Markov model	National health service	Lifetime (3.5%)	2018
Kim (2021) [40]	Australia	Mobile stroke unit^b^	Standard ambulance and hospital stroke care pathway	Cost-utility	DALY	Economic simulation model	Healthcare providers	5 years (5%)	2018
Morii (2021) [38]	Japan	Mobile interventionist^c^	Patients treated with EVT in hub facilities	Cost-utility	QALY	Simulation model	Government	3 years (2%)	2020
Bayer (2020) [39]	Singapore	A public information campaign to raise awareness of stroke symptoms and urgency (combined with other interventions)	Current practice	Cost-utility	QALY	Population-level systems dynamics model	Healthcare payer	15 years (3%)	NR
McMeekin (2019) [29]	England	30 EVT centers	24 EVT centers	Cost-utility	QALY	Discrete event simulation	Payer	5 years (NR)	2017
Stevens (2019) [23]	US	**Intervention 1:** enhanced educational material **Intervention 2:** interactive intervention^d^	Standard care	Cost-utility	Life year gain, QALY	Markov model	Societal and healthcare	5 years (3%)	2015
Whetten (2018) [26]	US	Access to critical cerebral emergency support services	Standard care	Cost-utility	QALY	Decision tree model	Healthcare payer	90 days (NR)	2015
Yan (2018) [27]	Canada	Combine different modes of transportation: -Mothership^e|^ by ground/ air -Drip-and-ship^f^ by ground/ air to minimum time to IVT -drip-and-ship by ground/air to minimum time to EVT	Mothership by ground	Cost-utility	QALY	Decision tree and Markov model	Payer	Lifetime (5%)	NR
Lahr (2017) [33]	Netherlands	**Intervention 1:** 10 stroke centers **Intervention 2:** 5 stroke centers + 5 hospitals without IVT **Intervention 3:** 3 stroke centers + 7 hospitals without IVT	Current situation: 9 community hospitals with IVT + 1 stroke center	Cost-effectiveness	Thrombolysis rate, OTT time, extra healthy life days	Discrete event simulation	Policymaker	NR (NR)	NR
Goff-Pronost (2017) [35]	France	8 SUs without teleconsultation	3 SUs and teleconsultation with emergency services in 5 hospitals	Cost-effectiveness	Thrombolysis rate	Decision tree-based analytical model	Hospital	1 year (NR)	NR
Espinoza (2017) [36]	Belgium	Standard stroke care supplemented with in-ambulance telemedicine	Standard stroke care	Cost-utility	QALY	Decision tree and Markov model	Healthcare payer	Lifetime (3% for costs and 1.5% for QALY)	2014
Torabi (2016) [25]	US	**Intervention 1:** telemedicine in outer-ring hospitals + stroke physician location at home **Intervention 2:** telemedicine in all hospitals + stroke physician location at home **Intervention 3:** no telemedicine + stroke physician location at center **Intervention 4:** telemedicine in outer-ring hospitals + physician location at center **Intervention 5:** telemedicine in all hospitals + physician location at center	No telemedicine + stroke physician location at home	Cost-effectiveness	OTT time, door-to-needle time, % of IVT within 3 h	Monte Carlo simulation model	NR	Lifetime (3%)	NR
Gyrd-Hansen (2015) [32]	Germany	Stroke emergency mobile	Normal EMS	Cost-utility	QALY	Economic evaluation along with clinical trial	Third-party payer	5 years (3%)	NR
Penaloza-Ramos (2014) [34]	England	**Intervention 1:** Divert GP calls ambulance service **Intervention 2:** Reduce time to call emergency service (series of educational interventions) **Intervention 3:** Immediate CT scan (CT scanner moved closer to the emergency department ward)	Current practice	Cost-utility	QALY	Decision tree model	National health service and Personal Social Services	Lifetime (3.5%)	2011
Dietrich (2014) [31]	Germany	Mobile stroke unit^†^	Normal EMS	Cost–benefit	Monetary benefit	1-year model	NR	1 year (NR)	NR
McMeekin (2013) [30]	England	Central provision of 2 regional IVT centers	10 local acute SUs	Cost-utility	QALY	Microsimulation (with Markov component)	NR	5 years (NR)	2011
Demaerschalk (2013) [22]	US	Hub-and-spoke telestroke network	No network between hub and spokes	Cost-utility	QALY	Markov model	Societal	Lifetime (3%)	2011
Switzer (2013) [24]	US	Hub-and-spoke telestroke network	No network between hub and spokes	Cost-effectiveness	Number of home discharges, inpatient rehabilitation/ nursing home discharges, in-hospital deaths	Decision analytic model	A network, a hub hospital, and a spoke hospital	5 years (3%)	2011

*CT* computed tomography, *DALY* disability-adjusted life year, *EMS* emergency medical services, *EVT* endovascular thrombectomy, *GP* general practitioner, *IVT* intravenous thrombolysis, *NR* not reported, *OTT* onset to treatment, *QALY* quality-adjusted life year, *SU* stroke unit

^a^Interventions included pre-notification of the in-hospital stroke treatment team, patient preparation during transport, direct transport to CT lab, delaying collection of blood

samples after intravenous thrombolysis administration for suitable patients, patient examination and administration of IVT bolus dose in the CT lab

^b^An ambulance equipped with a CT scanner, a point-of care laboratory, and telemedicine for early diagnosis

^c^Thrombectomy specialists (interventionists) travel to stroke centers closer to the patients to perform EVT

^d^Including educational materials, a medical alert bracelet, and in-hospital interactive group sessions (presentation, video, and roleplaying)

^e^Mothership model is direct transport patients to a comprehensive stroke center for IVT and EVT

^f^Drip-and-ship model is to first transport patients to a primary stroke centers for IVT. Next, when eligible, patients may be transported to the nearest comprehensive stroke center to undergo EVT

The majority of studies applied cost-utility analysis (n = 14) with quality-adjusted life year (QALY) (*n* = 13) [21–23, 26–30, 32, 34, 36, 38, 39] and disability-adjusted life year (DALY) (*n* = 1) [40] as the primary outcome of effectiveness. Two studies reported cost-effectiveness based on the IVT rate [29, 30], one study based on the number of home discharges [20], one study based on three outcome variables, including the IVT rate within three hours from symptom onset, OTT time, and door-to-needle time [25], and one study based on door-to-needle time, and death averted [37]. One study analyzed the cost–benefit of the used strategy [31].

Most economic evaluations conducted a model-based approach, while two studies were trial-based analyses [32, 37]. Simulation modeling was applied in six studies [25, 29, 30, 33, 38, 40], decision tree analytic modeling was used in four studies [24, 26, 34, 35], the use of a decision tree and a Markov model was adopted in four studies [21, 27, 28, 36], and two studies used a Markov model [22, 23]. Additionally, one study used a population-level systems dynamics model [39], and one study did not report a clear model [31].

Economic evaluations were analyzed from various perspectives containing a healthcare payer or payer perspective (*n* = 7) [26, 27, 29, 32, 36, 37, 39], a hospital or healthcare provider perspective (*n* = 3) [24, 35, 40], a national health service viewpoint (*n* = 2) [28, 34], a societal perspective (*n* = 3) [21–23], a policy maker viewpoint (*n* = 1) [33] and a government perspective (*n* = 1) [38]. Hence, most of studies included medical service costs, such as ambulance transportation, hospitalization, outpatient visits, rehabilitation, and long-term care. However, only two studies with a societal perspective reported indirect costs (productivity loss) [21, 23]. In three studies, no perspective was mentioned. One study included health care costs (ambulance service, treatment costs, bed day, nursing home, residential care home, assisted living facility, and carer visiting) [30], and two studies considered intervention costs and costs of different functional status after stroke [25, 31].

Time horizons ranged from 90 days to a lifetime. Seven studies used a 5-year horizon [23, 24, 29, 30, 32, 37, 40], and six studies employed a lifetime perspective [22, 25, 27, 28, 34, 36]. A ninety-day horizon was used in one study [26]. Discount rates were reported in 13 studies ranging from 1.5% to 5.0% [21–25, 27, 28, 32, 34, 36, 38–40]. Seven studies did not report any discount rate. Of those, the discount rate was not applicable for three studies due to a short time horizon of 90 days or 12 months [26, 31, 35].

### Quality of studies

Total CHEERS scores, along with percentage scores of included studies, are presented in Table 2. Fourteen studies were assessed as high quality (ranging from 79.2% to 93.8%) and six studies as medium quality (ranging from 66.7% to 72.9%). There were no studies that were assessed as low quality. The assessment on each item of the CHEERS statement is described in the Supplementary Information (Fig. S1).

**Table 2:** Reporting quality assessment

Study	CHEERS score							
	Title, abstract, and introduction (3)	Methods (14)	Results (4)	Discussion (1)	Other (2)	Total scores (24)	% of items scores	Reporting quality
Ajmi (2021) [37]	2.5	10.0	2.0	1.0	2.0	17.5	72.9	Medium
Tan (2021) [21]	2.5	14.0	3.0	1.0	2.0	22.5	93.8	High
Coughlan (2021) [28]	2.5	12.5	4.0	0.5	2.0	21.5	89.6	High
Kim (2021) [40]	2.5	11.0	2.5	1.0	2.0	19.0	79.2	High
Morii (2021) [38]	2.5	11.0	3.5	1.0	2.0	20.0	83.3	High
Bayer (2020) [39]	1.0	12.5	3.0	1.0	2.0	19.5	81.3	High
McMeekin (2019) [29]	2.5	12.0	3.0	0.5	2.0	20.0	83.3	High
Stevens (2019) [23]	2.5	13.0	3.5	1.0	2.0	22.0	91.7	High
Whetten (2018) [26]	2.5	11.5	3.0	1.0	2.0	20.0	83.3	High
Yan (2018) [27]	0.5	13.5	3.0	0.5	0.0	17.5	72.9	Medium
Lahr (2017) [33]	3.0	10.0	3.0	1.0	2.0	19.0	79.2	High
Goff-Pronost (2017) [35]	1.5	10.5	1.0	1.0	2.0	16.0	66.7	Medium
Espinoza (2017) [36]	2.5	13.0	3.0	0.5	1.0	20.0	83.3	High
Torabi (2016) [25]	2.0	9.0	3.0	1.0	2.0	16.0	66.7	Medium
Gyrd-Hansen (2015) [32]	2.0	11.0	4.0	1.0	2.0	20.0	83.3	High
Penaloza-Ramos (2014) [34]	2.5	12.5	3.0	1.0	2.0	21.0	87.5	High
Dietrich (2014) [31]	2.5	9.0	4.0	1.0	0.0	16.5	68.8	Medium
McMeekin (2013) [30]	1.5	10.5	2.5	1.0	2.0	17.5	72.9	Medium
Demaerschalk (2013) [22]	2.5	13.0	2.0	0.5	2.0	20.0	83.3	High
Switzer (2013) [24]	2.5	12.5	2.5	1.0	2.0	20.5	85.4	High

High: % of items scores is 75.0% or more; medium: % of items scores is from 50.0% to 74.9%; low: % of items scores is less than 50.0%

### Results of economic evaluation

Included studies were categorized into four main groups based on the type of interventions: (1) educational interventions, (2) organizational models, (3) healthcare delivery infrastructure, and (4) workflow improvements (see Table 3).

**Table 3:** Study results

Study	Intervention vs. comparator	Incremental costs	Incremental effectiveness	ICER^a^	Threshold	Conclusion
**Educational interventions**						
Bayer (2020) [39]	Public information campaign + IVT vs. CP	SD$7,000,000	1741 QALYs	SD$4,021/QALY	SD$70,000	Cost-effective
	Public information campaign + IVT + EVT vs. CP	SD$33,000,000	2121 QALYs	SD$15,559/QALY		
	Public information campaign + other five interventions vs. CP	SD$402,000,000	9411 QALYs	SD$ 42,716/QALY		
Stevens (2019) [23]	Enhanced educational material vs. SC	$11.85	0.00014 QALYs	$84,643/QALY	$100,000	Cost-effective (II)
	Interactive intervention (II) vs. SC	$10.04	0.00017 QALYs	$59,508/QALY		
Penaloza-Ramos (2014) [34]	GP staff are trained to better recognize stroke vs. CP	-$32,305	2.26 QALYs	-	$30,000	Dominant
	Reduce time to call emergency service (series of educational interventions) vs. CP	-$16,153	1.14 QALYs	-		Dominant
**Organizational models**						
Tan (2021) [21]	Telemedicine vs. no telemedicine	-$627	0.0925 QALYs	-	$27,736	Dominant
Morii (2021) [38]	Mobile interventionist vs. CP in Kamikawachubu	NR	4.33 QALYs	$12,572/QALY	$48,146	Cost-effective
	Mobile interventionist vs. CP in HoKumo	NR	1.58 QALYs	$89,899/QALY		Not cost-effective
	Mobile interventionist vs. CP in Sapporo	NR	NR	$969,766/QALY		
	Mobile interventionist vs. CP in Nakasorachi	NR	NR	1,634,636/QALY		
	Mobile interventionist vs. CP in Nishiiburi	NR	NR	$567,511/QALY		
	Mobile interventionist vs. CP in Tokachi	NR	NR	$1,078,899/QALY		
	Mobile interventionist vs. CP in Kushiro	NR	NR	$1,438,215/QALY		
Kim (2021) [40]	Mobile stroke unit vs. standard ambulance and hospital stroke care pathway	AU$1,389,159	44.84 DALY avoided	AU$30,982/ DALY avoided	AU$50,000	Cost-effective
Yan (2018) [27]	Optimal model (Mothership by ground/air + drip-and-ship by ground/air) vs. mothership by ground	-CA$2,035	0.023QALYs	-	CA$50,000	Dominant
Whetten (2018) [26]	Access to critical cerebral emergency support services vs. SC	-$4,241	0.2 QALYs	-	NR	Dominant
Espinoza (2017) [36]	SC supplemented with in-ambulance telemedicine vs. SC	-$4,040	4.9 QALYs	-	$47,747	Dominant
Dietrich (2014) [31]	Mobile stroke unit vs. regular EMS	$208,629	$408,429	1.96^b^	1	Cost-effective
Torabi (2016) [25]	Telemedicine in outer-ring hospitals + stroke physician location at home vs. CP (none telemedicine + stroke physician location at home)	-$114,500	4.9% IVT within 3 h	-	NR	Dominant
	Telemedicine in all hospitals + stroke physician location at home vs. CP	-$242,500	5.2%	-	NR	Dominant
	None telemedicine + stroke physician location at center vs. CP	-$72,500	3.7%	-	NR	Dominant
	Telemedicine in outer-ring hospitals + physician location at center vs. CP	-$180,750	8.1%	-	NR	Dominant
	All telemedicine + physician location at center vs. CP	-$242,500	10.5%	-	NR	Dominant
Gyrd-Hansen (2015) [32]	Stroke emergency mobile vs. regular EMS	€963,295	29.7 QALYs	€32,456/ QALY	NR €36,223^c^	Cost-effective
McMeekin (2013) [30]	Redirection to 2 regional neuroscience centers vs. 10 local acute stroke units	£6,730	12.6 QALYs	£534/QALY	NR £29,594^d^	Cost-effective
Demaerschalk (2013) [22]	Hub-and-spoke telestroke network vs. no network between hub and spokes	-$1,436	0.02 QALYs	-	$50,000	Dominant
Switzer (2013) [24]	Hub-and-spoke telestroke network vs. no network between hub and spokes	-$358,435	6.11 more patients discharged home per year	-	NR	Dominant
**Healthcare delivery infrastructure**						
McMeekin (2019) [29]	30 EVT centers vs. 24 EVT centers	-£2,870,000	213 QALYs	-	£30,000	Dominant
Lahr (2017) [33]	Improving stroke care at 9 community hospitals to stroke center vs. CP (9 community hospitals with thrombolysis)	$912	8.0% thrombolysis rate	$113/ 1% thrombolysis rate	NR	NR
	Centralization and improve stroke care in 4 hospitals, 5 hospitals without thrombolysis vs. CP	$540	7.4% thrombolysis rate	$71/ 1% thrombolysis rate		
	Centralization and improve stroke care in 2 hospitals and 7 hospitals without thrombolysis vs. CP	$395	6.8% thrombolysis rate	$56/ 1% thrombolysis rate		
Goff-Pronost (2017) [35]	8 stroke units (without telemedicine) vs. current situation (3 stroke units and teleconsultation with emergency services in 5 hospitals)	$264	0.4% thrombolysis rate	$660/ 1% thrombolysis rate	NR	NR
**Workflow improvement**						
Ajmi (2021) [37]	Quality improvement project, included streamlining stroke care pathway and simulation-based training vs. no quality improvement project	NR	NR	$29/ minute door-to-needle time reduction $10,543/death averted	$43,000/QALY	NR
Coughlan (2021) [28]	Inter-hospital transfer by helicopter vs. ground EMS	£3,785	0.14 QALYs	£28,027/QALY	£ 30,000	Cost-effective (helicopter)
Penaloza-Ramos (2014) [34]	Immediate CT scan (e.g. CT scanner moved closer to the emergency department ward) vs. CP	-$24,963	1.81 QALYs	-	$30,000	Dominant

*Ca$* Canadian Dollar, *CP* current practice, *CT* computed tomography, *DALY* disability-adjusted life year, *EMS* emergency medical services, *EVT* endovascular thrombectomy, *GP* general practitioner, *IVT* intravenous thrombolysis, *NR* not reported, *QALY* quality-adjusted life year, *SC* standard care, *SD$* Singapore Dollar

^a^ ICER (incremental cost-effectiveness ratio) is calculated by dividing the different costs by the different effectiveness of two strategies

^b^Benefit-cost ratio

^c^1GDP per capita in Germany in 2014 [41, 42]

^d^1GDP per capita in the UK in 2011 [43]

#### Educational interventions

Three studies assessed the cost-utility of educational interventions to reduce time delays to treatment in AIS patients [23, 34, 39]. Topics varied across studies, including public campaigns, interactive interventions for patients (i.e. educational materials, medical alert bracelets, and in-hospital interactive group sessions), and training staff of general practitioner offices. Irrespective of the type of intervention, all educational interventions were cost-effective as their ICERs were lower than the willingness-to-pay thresholds. One study even demonstrated that educational interventions for both health staff and patients were dominant compared to current practice (higher QALY gained (1.14- 2.26 QALYs) and saving $16,153- $32,305) [34].

#### Organizational models

Over half of the selected studies (12/20) performed economic evaluations on organizational models. Different strategies were considered, including studies on telemedicine solutions between stroke centers and community hospitals [21, 22, 24–26], in-ambulance telemedicine [36], mobile stroke units [31, 32, 40], a combination of mothership model and drip-and-ship model with alternative transportation modes (ground or air) [27], prehospital redirection of patients to regional IVT center instead of local stroke units [30], and a mobile interventionist [38]. Most of these studies (11 out of 12) reported that interventions were cost-effective or dominant (higher QALY gained and saving costs) [22, 24–27, 30–32, 36, 40]. However, one Japanese study reporting on the use of a mobile interventionist showed that this approach was only cost-effective in a specific region (i.e. Kamikawachubu area in Hokkaido). In Hokkaido, the ICER of mobile interventionists was higher than the threshold of $48,146 in other areas [38].

#### Healthcare delivery infrastructure

Three economic evaluations reported interventions related to the regional healthcare delivery infrastructure for acute stroke care, such as introducing new EVT centers [29], upgrading hospitals to IVT-capable stroke care centers [33, 35] and centralization of acute stroke care treatment [33]. Effectiveness was measured as the percentage of the IVT rate in two studies. Within these studies, the ICER ranged from $56 to $660 per one percentage increase in the IVT rate [33, 35]. One study showed that adding new EVT-capable centers in their region gained an additional 213 QALYs and saved £2,870,000 compared to the current situation [29].

#### Workflow improvement

Three studies assessed economic evaluations of workflow improvements [28, 34, 37]. Implementing a quality improvement project, including streamlining stroke care pathway and simulation-based training, produced an additional $29 per minute door-to-needle time reduction and $10,543 per death averted [37]. One study evaluated inter-hospital transfer by helicopter emergency medical service compared to ground emergency medical service, which appeared cost-effective at the threshold of £30,000 per QALY [28]. Another study demonstrated that an immediate computed tomography (CT) scan strategy, in which the CT scanner was moved closer to the emergency department, was dominant when compared to current practice [34].

**Fig. 2:**
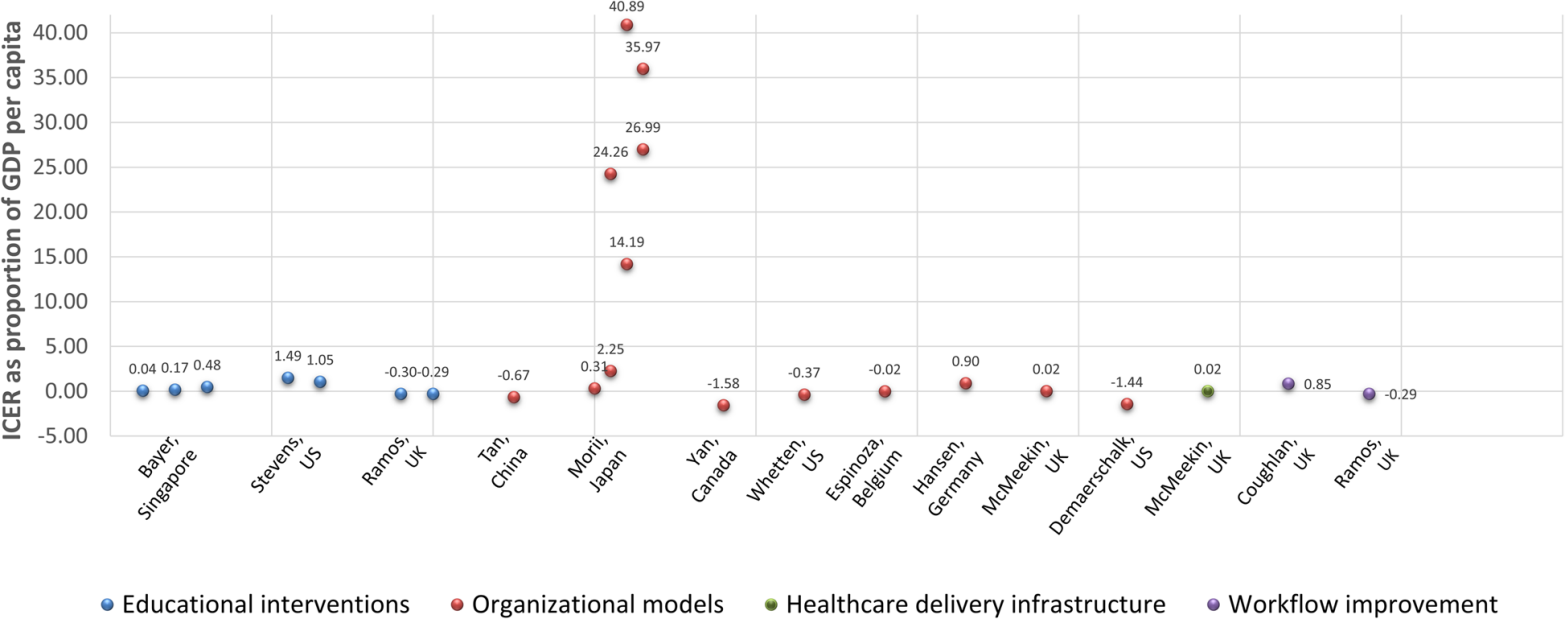
ICERs compared to country GDP per capita

The ICERs of studies reporting QALYs (*n* = 13) [21–23, 26–30, 32, 34, 36, 38, 39] were assessed as a proportion of country GDP per capita (Fig. 2). Cross-study analysis indicated that the strategies of educational interventions, organizational models, healthcare delivery infrastructure, and workflow improvement were highly cost-effective with the ICER less than one times GDP per capita (ranging from -1.58 to 0.85 times GDP per capita) in 11 studies. Stevens et al. showed that the educational interventions in the US exceeded one times GDP (1.05 to 1.49 times GDP per capita). However, organizational models in Japan were not considered cost-effective in most of regions with the ICERs ranging from 2.25 to 40.89 times GDP per capita.

Three studies had more than 1 intervention (3 interventions in Bayer’s study, 2 interventions in Stevens’ study, 3 interventions in Ramos’ study); 1 intervention in 7 regions in Morii’s study. ICER: incremental cost-effectiveness ratio.

The majority of included studies (18/20) performed sensitivity analysis. Of these studies, probabilistic sensitivity analysis was conducted in seven studies. More information on the sensitivity analyses is summarized in the Supplementary Information (Table 1).

## Discussion

### Principal findings

To our knowledge, this is the first systematic review on the cost-effectiveness of strategies aimed to reduce OTT time for AIS patients. Twenty studies met the eligibility criteria and were included in this systematic review. Based on factors associated with time reductions along the acute stroke care pathway [9, 11, 44], we categorized strategies into four groups: educational interventions, organizational models, healthcare delivery infrastructure and workflow improvements. The results of this review demonstrate that all intervention types may be cost-effective or even dominant when compared with current stroke care practices [21–29, 31, 34, 36, 39, 40]. Two studies in ‘organizational model’ category [30, 32], in which the willingness to pay threshold was not stated, were deemed cost-effective if the threshold of one GDP per capita was applied [17]. Accordingly, these strategies should be considered as interventions to optimize acute stroke care systems, as they improve functional outcomes for AIS patients and reduce long-term costs of disability after stroke. However, some improvement approaches might be region-specific, as Morii et al. [38] found that a mobile interventionist strategy was cost-effective in only one area (i.e. Kamikawahokubu) while not feasible in other areas due to the close distance between the hub and spoke centers and a relatively low incidence of LVO patients. We could not conclude whether interventions were cost-effective for two studies [33, 35] in the category ‘healthcare delivery infrastructure’ and for one study [37] in the category ‘workflow improvement’ because the primary outcome was not measured in QALYs. In addition, cross-study analysis of studies reporting QALYs showed that all intervention types were highly cost-effective in most of countries (11/13 studies).

In our review, 70% of included studies were classified as high quality based on the CHEERS score. The majority of the studies referred to a cost-utility analysis, in which the study design (i.e. the perspective, time horizon, and models) differed across studies. However, most of studies (16/20) showed that strategies aimed at reducing the OTT time were cost-effective. Although strategies were categorized into four groups, classifying articles was not straightforward because various strategies were simultaneously considered in economic evaluations. For example, Penaloza-Ramos et al. conducted health economic evaluations of seven strategies simultaneously, including health staff training (in the ‘educational intervention’ category) and timely CT scan (in the ‘workflow improvement’ category) [34]. Bayer et al. performed the cost-effectiveness analysis of combining a public information campaign with five other interventions, such as IVT and EVT treatment in the acute stroke unit, out-of-hospital rehabilitation, and secondary prevention. The results showed that combining interventions was the most cost-effective strategy [39].

### Policy relevance

Incorporating the cost-effectiveness evidence, apart from the current set-up of regional stroke services, other local factors need to be taken into account before intervening in regional stroke care pathways, such as the incidence of stroke, population density, and geographic location of stroke centers. From a variety of possible improvement strategies, policymakers and other stakeholders will have to identify the ones most favorable in terms of effects and cost implications. For example, the mobile interventionist strategy is cost-effective in the specific area with a high incidence of LVO, more than three-hour travel for patients to a hub (intervention) center, and within one-hour travel for interventionists [38]. Furthermore, Yan et al. identified the optimal strategy in delivering EVT, as the combination of both mothership model and drip-and-ship model based on the geographical location of stroke onset and stroke centers [27]. As such, it can be inferred that multiple organizational models need to be considered in stroke management according to local characteristics.

### Strengths and limitations

The strength of our systematic review is in its wide scope, providing a broad range of various strategies to reduce time delay compared with focusing on one or more specific interventions. Additionally, all types of full economic evaluations, with both trial- and model-based analysis, were included in this review. Furthermore, it provides an informative synthesis of the cost-effectiveness evidence for all strategies, enabling better-informed decisions in stroke care, especially in the context of constrained health resources and the ageing population.

Nevertheless, several limitations in the review should be noted. Firstly, direct comparison between strategies was not possible due to heterogeneity across studies, such as setting, effectiveness outcomes, and comparators. Therefore, a ranking and assessment could not be offered. The latter would be difficult, as generic weighing and balancing are impractical without taking region-specific characteristics of the local setting into account. Instead, we offer a narrative synthesis which will help local policymakers to consider their individual options. Another limitation is to include only English papers in the present systematic review. However, no language restriction was applied in our search strategy, and screening title and abstract revealed that non-English articles also met other exclusion criteria. Finally, the results of this review were based on published studies only, thus potentially leading to publication bias.

### Future perspective and recommendation

Our study demonstrates the cost-effectiveness of various strategies aiming at reducing OTT, but other organizational strategies are currently lacking a cost-effectiveness perspective. For example, the use of prehospital triage tools to transfer stroke patients directly to the appropriate target hospitals [45] or workflow improvements in which suspected LVO patients are directly transferred to the angiography suite [46] may be efficient solutions to further reduce time delays to EVT initiation. This emphasizes the need of economic evaluations for these strategies.

There is also a notable gap persisting in the cost-effectiveness analysis of strategies reducing OTT time in low- or middle-income countries. In such settings policymakers and health care providers are possibly in the phase of building infrastructure without the opportunity to consider alternative organizational set-ups (i.e. mobile stroke unit, mobile interventionist) [47]. Although stroke remains a major cause of death and disability worldwide, stroke incidence and mortality rates recently have shown to decline in high-income countries due to improvements in primary and secondary prevention as well as in acute reperfusion treatments [48]. However, the trend in low- and middle-income countries is the opposite, resulting in a significant stroke burden in these regions [49]. Given the concerning shift in stroke epidemiology to low- or middle-income countries, important choices lie ahead, even when considering the very basic infrastructure.

Current economic evaluations use healthcare or payer perspectives that do not include indirect costs of post-stroke, such as productivity loss and informal caregiving for stroke patients. However, indirect costs of post-stroke account for approximately 33% of the total economic burden of stroke [50]. There is a remarkable increase worldwide in stroke incidence among younger groups (i.e. less than 65 years old) [51], which is likely to increase the indirect cost of stroke burden. Hence, future studies need to take into account the lack of evidence of societal perspective, and in low-and middle-income countries.

## Conclusions

The results of this systematic review show that reported strategies reducing time delay in stroke care services are mostly cost-effective across different settings. While the findings from this review provide mainly positive results, local characteristics and background of regional health care systems should be taken into account.

All authors were involved with the conception and study design. Chi Phuong Nguyen and Willemijn J Maas performed screening records, data extraction, and data analysis. Maarten M H Lahr was the third reviewer. Chi Phuong Nguyen outlined the manuscript. Chi Phuong Nguyen and Willemijn J Maas drafted the manuscript. All authors critically reviewed the entire manuscript and approved the final submission.

## Supplementary Information

**Additional file 1: Appendix 1. **Search strategy description. **Supplementary Table S1. **Sensitivity analysis of included studies. **Supplementary Fig. S1. **Quality assessment of included studies according to each item of the CHEERS statement. **Supplementary Table S2. **PRISMA checklist. **Supplementary Table S3.**PRISMA 2020 for Abstract Checklist.

## Data Availability

All data generated or analyzed during this study are included in this published article and its supplementary information files.

## Abbreviations

AIS: Acute ischemic stroke
CHEERS: Consolidated Health Economic Evaluation Reporting Standards
DALY: Disability-adjusted life year
EVT: Endovascular thrombectomy
GDP: Gross Domestic Product
ICER: Incremental cost-effectiveness ratio
IVT: Intravenous thrombolysis
LVO: Large vessel occlusion
OTT: Onset to treatment
QALY: Quality-adjusted life year
