# Supplementary material for: Cost-effectiveness of improvement strategies for reperfusion treatments in acute ischemic stroke: a systematic review
Source: BMC Health Serv Res. 2023 Mar 30;23:315. doi: 10.1186/s12913-023-09310-0 (PMC10064746; doi:10.1186/s12913-023-09310-0)
Supplement: Supplementary file 1 — Additional file 1: Appendix 1. Search strategy description. Supplementary Table S1. Sensitivity analysis of included studies. Supplementary Fig. S1. Quality assessment of included studies according to each item of the CHEERS statement. Supplementary Table S2. PRISMA checklist. Supplementary Table S3.PRISMA 2020 for Abstract Checklist. [file 12913_2023_9310_MOESM1_ESM.docx]

**Supplementary Information**

**Cost-effectiveness of improvement strategies for reperfusion treatments in acute ischemic stroke: a systematic review**

Chi Phuong Nguyen^1,2,3*^, Willemijn J Maas^2,4*^, Durk-Jouke van der Zee^1,2^, Maarten Uyttenboogaart^4,5^, Erik Buskens^1,2^, Maarten M H Lahr^2^, on behalf of the CONTRAST consortium

^1^ Department of Operations, Faculty of Economics and Business, University of Groningen, Groningen, the Netherlands

^2^ Department of Epidemiology, University of Groningen, University Medical Center Groningen, the Netherlands

^3^ Department of Pharmaceutical Administration and Economics, Hanoi University of Pharmacy, Hanoi, Vietnam

^4^ Department of Neurology, University of Groningen, University Medical Center Groningen, the Netherlands

^5^ Department of Radiology, Medical Imaging Center, University of Groningen, University Medical Center Groningen, the Netherlands

**These authors contributed equally to this work*

Corresponding author: Chi Phuong Nguyen

Email: [p.c.nguyen@rug.nl](mailto:p.c.nguyen@rug.nl)

**Appendix 1.** Search strategy description

**PubMed (access on June 30, 2021)**

**#1** ("Stroke"[Mesh] OR stroke*[tiab] OR “cerebrovascular accident*”[tiab] OR CVA[tiab] OR CVAs[tiab] OR “cerebrovascular event*”[tiab] OR “brain vascular accident*”[tiab] OR “brain infarc*”[tiab] OR “brain ischemi*”[tiab] OR “brain attack*”[tiab] OR “brain accident*”[tiab] OR “cerebral infarc*”[tiab] OR “cerebral ischemi*”[tiab] OR “cerebral vessel occlusion*”[tiab] OR “large vessel occlusion*”[tiab] OR “intracranial ischemi*”[tiab] OR “intracranial infarction*”[tiab] OR “intracranial vessel occlusion*”[tiab] OR “brain vessel occlusion*”[tiab]) ***(***[***342,995***](https://pubmed.ncbi.nlm.nih.gov/?term=%28%22Stroke%22%5BMesh%5D+OR+stroke%2A%5Btiab%5D+OR+%E2%80%9Ccerebrovascular+accident%2A%E2%80%9D%5Btiab%5D+OR+CVA%5Btiab%5D+OR+CVAs%5Btiab%5D+OR+%E2%80%9Ccerebrovascular+event%2A%E2%80%9D%5Btiab%5D+OR+%E2%80%9Cbrain+vascular+accident%2A%E2%80%9D%5Btiab%5D+OR+%E2%80%9Cbrain+infarc%2A%E2%80%9D%5Btiab%5D+OR+%E2%80%9Cbrain+ischemi%2A%E2%80%9D%5Btiab%5D+OR+%E2%80%9Cbrain+attack%2A%E2%80%9D%5Btiab%5D+OR+%E2%80%9Cbrain+accident%2A%E2%80%9D%5Btiab%5D+OR+%E2%80%9Ccerebral+infarc%2A%E2%80%9D%5Btiab%5D+OR+%E2%80%9Ccerebral+ischemi%2A%E2%80%9D%5Btiab%5D+OR+%E2%80%9Ccerebral+vessel+occlusion%2A%E2%80%9D%5Btiab%5D+OR+%E2%80%9Clarge+vessel+occlusion%2A%E2%80%9D%5Btiab%5D+OR+%E2%80%9Cintracranial+ischemi%2A%E2%80%9D%5Btiab%5D+OR+%E2%80%9Cintracranial+infarction%2A%E2%80%9D%5Btiab%5D+OR+%E2%80%9Cintracranial+vessel+occlusion%2A%E2%80%9D%5Btiab%5D+OR+%E2%80%9Cbrain+vessel+occlusion%2A%E2%80%9D%5Btiab%5D%29&ac=no&sort=relevance)***)***

**#2** ("Thrombectomy"[Mesh] OR "Thrombolytic Therapy"[Mesh] OR "Tissue Plasminogen Activator"[Mesh] OR "Reperfusion"[Mesh] OR thrombectom*[tiab] OR EVT[tiab] OR endovascular[tiab] OR IVT[tiab] OR thrombo*[tiab] OR fibrinoly*[tiab] OR “plasminogen activator*”[tiab] OR alteplase[tiab] OR tenecteplase[tiab] OR reperfusion[tiab] OR “acute stroke care”[tiab] OR “acute stroke treatment*”[tiab]) ***(***[***577,561***](https://pubmed.ncbi.nlm.nih.gov/?term=%28%22Thrombectomy%22%5BMesh%5D+OR+%22Thrombolytic+Therapy%22%5BMesh%5D+OR+%22Tissue+Plasminogen+Activator%22%5BMesh%5D+OR+%22Reperfusion%22%5BMesh%5D+OR+thrombectom%2A%5Btiab%5D+OR+EVT%5Btiab%5D+OR+endovascular%5Btiab%5D+OR+IVT%5Btiab%5D+OR+thrombo%2A%5Btiab%5D+OR+fibrinoly%2A%5Btiab%5D+OR+%E2%80%9Ctissue+plasminogen+activator%E2%80%9D%5Btiab%5D+OR+alteplase%5Btiab%5D+OR+tenecteplase%5Btiab%5D+OR+reperfusion%5Btiab%5D+OR+%E2%80%9Cacute+stroke+care%E2%80%9D%5Btiab%5D+OR+%E2%80%9Cacute+stroke+treatment%2A%E2%80%9D%5Btiab%5D%29&ac=no&sort=relevance)***)***

**#3** ("Health Services Administration"[Mesh] OR "Time-to-Treatment"[Mesh] OR “Time Factors”[Mesh]

OR "Regional Health Planning/organization and administration"[Mesh] OR "Delivery of Health Care, Integrated/economics"[Mesh] OR "Ambulatory Care Facilities/economics"[Mesh] OR "Ambulatory Care Facilities/organization and administration"[Mesh] OR “Emergency Medical Services”[Mesh] OR “Workflow”[Mesh] OR “health service*”[tiab] OR time*[tiab] OR “regional health plan*”[tiab] OR “health care plan*”[tiab] OR “delivery*”[tiab] OR ambula*[tiab] OR “organizational model*”[tiab] OR “geographic model*”[tiab] OR reconfigur*[tiab] OR (drip[tiab] *AND* ship[tiab]) OR mothership*[tiab] OR (trip[tiab] *AND* treat[tiab]) OR “mobile stroke unit*”[tiab] OR MSU [tiab] OR “mobile unit*”[tiab] OR “mobile CT unit*”[tiab] OR (drip[tiab] *AND* drive [tiab]) OR (drive[tiab] *AND* retrieve [tiab]) OR transfer*[tiab] OR transport*[tiab] OR dispatch*[tiab] OR “primary stroke cent*”[tiab] OR “comprehensive stroke cent*”[tiab] OR “EVT cent*”[tiab] OR “EVT-capable cent*”[tiab] OR delay*[tiab] OR prehospital[tiab] OR “pre-hospital”[tiab] OR “stroke alert”[tiab] OR emergenc*[tiab] OR triage[tiab] OR “hub-and-spoke model”[tiab] OR “hub cent*”[tiab] OR “spoke cent*”[tiab] OR telemedicine[tiab] OR train*[tiab] OR educat*[tiab] OR “pre-notification”[tiab] OR prenotification[tiab] OR workflow*[tiab] OR “work flow*”[tiab]) ***(9,901,099)***

**#4** ("Cost-Benefit Analysis"[Mesh] OR “cost effectiveness”[tiab] OR “cost utilit*”[tiab] OR “cost benefit*”[tiab] OR “economic outcome*”[tiab] OR “economic evaluation*”[tiab] OR “economic impact*”[tiab] OR “health economic*”[tiab] OR “economic modelling”[tiab] OR “economic assessment*”[tiab] OR “Quality-Adjusted Life Years”[Mesh] OR “quality-adjusted life year*”[tiab] OR QALY* [tiab] OR ICER[tiab] OR “incremental cost effectiveness ratio”[tiab]) ***(***[***151,474***](https://pubmed.ncbi.nlm.nih.gov/?term=%28%22Cost-Benefit+Analysis%22%5BMesh%5D+OR+%E2%80%9Ccost+effectiveness%E2%80%9D%5Btiab%5D+OR+%E2%80%9Ccost+utilit%2A%E2%80%9D%5Btiab%5D+OR+%E2%80%9Ccost+benefit%2A%E2%80%9D%5Btiab%5D+OR+%E2%80%9Ceconomic+outcome%2A%E2%80%9D%5Btiab%5D+OR+%E2%80%9Ceconomic+evaluation%2A%E2%80%9D%5Btiab%5D+OR+%E2%80%9Ceconomic+impact%2A%E2%80%9D%5Btiab%5D+OR+%E2%80%9Chealth+economic%2A%E2%80%9D%5Btiab%5D+OR+%E2%80%9Ceconomic+modelling%E2%80%9D%5Btiab%5D+OR+%E2%80%9Ceconomic+assessment%2A%E2%80%9D%5Btiab%5D+OR+%E2%80%9CQuality-Adjusted+Life+Years%E2%80%9D%5BMesh%5D+OR+%E2%80%9Cquality-adjusted+life+year%2A%E2%80%9D%5Btiab%5D+OR+QALY%2A+%5Btiab%5D+OR+ICER%5Btiab%5D+OR+%E2%80%9Cincremental+cost+effectiveness+ratio%E2%80%9D%5Btiab%5D%29&ac=no&sort=relevance)***)***

**#1 AND #2 AND #3 AND #4 *(466)***

**Filters: from 2010 to 2021 *(344)***

**EMBASE (access on June 30, 2021)**

**#1** ‘cerebrovascular accident’/exp OR (‘stroke*’ OR ‘cerebrovascular accident*’ OR ‘CVA’ OR ‘CVAs’ OR ‘cerebrovascular event*’ OR ‘brain vascular accident*’ OR ‘brain infarc*’ OR ‘brain ischemi*’ OR ‘brain attack’ OR ‘brain accident*’ OR ‘cerebral infarc*’ OR ‘cerebral ischemi*’ OR ‘cerebral vessel occlusion*’ OR ‘large vessel occlusion*’ OR ‘intracranial ischemi*’ OR ‘intracranial infarction*’ OR ‘intracranial vessel occlusion*’ OR ‘brain vessel occlusion*’):ab,ti ***(576,946)***

**#2** ‘thrombectomy’/exp OR ‘fibrinolytic therapy’/exp OR ‘plasminogen activator’/exp OR ‘reperfusion’/exp OR (‘thrombectom*’ OR ‘EVT’ OR ‘endovascular’ OR ‘IVT’ OR ‘thrombo*’ OR ‘fibrinoly*’ OR ‘alteplase’ OR ‘tenecteplase’ OR ‘plasminogen activator*’ OR ‘reperfusion’ OR ‘acute stroke care’ OR ‘acute stroke treatment*’):ab,ti ***(845,582)***

**#3** ‘health service’/exp OR ‘time to treatment’/exp OR ‘time factor’/exp OR ‘health care planning’/exp OR ‘health care delivery’/exp OR ‘workflow’/exp OR

(‘health service*’ OR ‘regional health plan*’ OR ‘health care plan*’ OR ‘delivery*’ OR ‘ambula*’ OR ‘organizational model*’ OR ‘geographic model*’ OR (‘drip’ AND ‘ship’) OR ‘mothership*’ OR (‘trip’ AND ‘treat’) OR ‘mobile stroke unit*’ OR ‘MSU’ OR ‘mobile unit*’ OR ‘mobile CT unit’ OR (‘drip’ *AND* ‘drive’) OR (‘drive’ *AND* ‘retrieve’) OR ‘transfer*’ OR ‘transport*’ OR ‘dispatch*’ OR ‘primary stroke cent*’ OR ‘comprehensive stroke cent*’ OR ‘EVT cent*’ OR ‘EVT-capable cent*’ OR ‘time*’ OR ‘delay*’ OR ‘prehospital’ OR ‘pre-hospital’ OR ‘stroke alert’ OR ‘emergenc*’ OR ‘triage’ OR ‘hub-and-spoke model’ OR ‘hub cent*’ OR ‘spoke cent*’ OR ‘telemedicine’ OR ‘train*’ OR ‘educat*’ OR ‘pre-notification’ OR ‘prenotification’ OR ‘workflow*’ OR ‘work flow*’):ab,ti ***(13,050,643)***

**#4** ‘cost benefit analysis’/exp OR ‘cost effectiveness analysis’/exp OR ‘cost utility analysis’/exp OR ‘quality adjusted life year’/exp OR (‘cost effectiveness’ OR ‘cost utilit*’ OR ‘cost benefit*’ OR ‘economic outcome*’ OR ‘economic evaluation*’ OR ‘economic impact*’ OR ‘health economic*’ OR ‘economic modelling’ OR ‘economic assessment*’ OR ‘quality adjusted life year*’ OR ‘QALY*’ OR ‘ICER’ OR ‘incremental cost effectiveness ratio’):ab,ti ***(298,480)***

**# 1 AND #2 AND #3 AND #4 *(1,222)***

**Filters: from 2010 to 2021 *(891)***

**Excluding conference abstract, letter, and chapter *(580)***

**Web of Science (access on June 16, 2021)**

**#1** TS=(stroke OR ‘cerebrovascular accident’ OR CVA OR CVAs OR ‘cerebrovascular event’ OR ‘brain vascular accident’ OR ‘brain infarc*’ OR ‘brain ischemi*’ OR ‘brain attack’ OR ‘brain accident*’ OR ‘cerebral infarc*’ OR ‘cerebral ischemi*’ OR ‘cerebral vessel occlusion’ OR ‘large vessel occlusion’ OR ‘intracranial ischemi*’ OR ‘intracranial infarction’ OR ‘intracranial vessel occlusion’ OR ‘brain vessel occlusion’) ***(474,987)***

**#2** TS=(‘thrombectom*’ OR ‘EVT’ OR ‘endovascular’ OR ‘IVT’ OR ‘thrombo*’ OR ‘fibrinoly*’ OR ‘alteplase’ OR ‘tenecteplase’ OR ‘plasminogen activator*’ OR ‘reperfusion’ OR ‘acute stroke care’ OR ‘acute stroke treatment*’) ***(657,847)***

**#3** TS=(‘health service*’ OR ‘regional health plan*’ OR ‘health care plan*’ OR ‘delivery*’ OR ‘ambula*’ OR ‘organizational model*’ OR ‘geographic model*’ OR (‘drip’ AND ‘ship’) OR ‘mothership*’ OR (‘trip’ AND ‘treat’) OR ‘mobile stroke unit*’ OR ‘MSU’ OR ‘mobile unit*’ OR ‘mobile CT unit’ OR (‘drip’ *AND* ‘drive’) OR (‘drive’ *AND* ‘retrieve’) OR ‘transfer*’ OR ‘transport*’ OR ‘dispatch*’ OR ‘primary stroke cent*’ OR ‘comprehensive stroke cent*’ OR ‘EVT cent*’ OR ‘EVT-capable cent*’ OR ‘time*’ OR ‘delay*’ OR ‘prehospital’ OR ‘pre-hospital’ OR ‘stroke alert’ OR ‘emergenc*’ OR ‘triage’ OR ‘hub-and-spoke model’ OR ‘hub cent*’ OR ‘spoke cent*’ OR ‘telemedicine’ OR ‘train*’ OR ‘educat*’ OR ‘pre-notification’ OR ‘prenotification’ OR ‘workflow*’ OR ‘work flow*’) ***(12,800,392)***

**#4** TS=(‘cost effectiveness’ OR ‘cost utilit*’ OR ‘cost benefit*’ OR ‘economic outcome*’ OR ‘economic evaluation*’ OR ‘economic impact*’ OR ‘health economic*’ OR ‘economic modelling’ OR ‘economic assessment*’ OR ‘quality adjusted life year*’ OR ‘QALY*’ OR ‘ICER’ OR ‘incremental cost effectiveness ratio’) ***(757,956)***

**#1 AND #2 AND #3 AND #4 (1,187)**

**Filters: from 2010 to 2021 (879)**

**Excluding meeting abstract, book chapter (866)**

**Additional search**

**Pubmed (From July 1^st^ until January 9^th^ 2022)**

**#1** ("Stroke"[Mesh] OR stroke*[tiab] OR “cerebrovascular accident*”[tiab] OR CVA[tiab] OR CVAs[tiab] OR “cerebrovascular event*”[tiab] OR “brain vascular accident*”[tiab] OR “brain infarc*”[tiab] OR “brain ischemi*”[tiab] OR “brain attack*”[tiab] OR “brain accident*”[tiab] OR “cerebral infarc*”[tiab] OR “cerebral ischemi*”[tiab] OR “cerebral vessel occlusion*”[tiab] OR “large vessel occlusion*”[tiab] OR “intracranial ischemi*”[tiab] OR “intracranial infarction*”[tiab] OR “intracranial vessel occlusion*”[tiab] OR “brain vessel occlusion*”[tiab]) ***(15,823)***

**#2** ("Thrombectomy"[Mesh] OR "Thrombolytic Therapy"[Mesh] OR "Tissue Plasminogen Activator"[Mesh] OR "Reperfusion"[Mesh] OR thrombectom*[tiab] OR EVT[tiab] OR endovascular[tiab] OR IVT[tiab] OR thrombo*[tiab] OR fibrinoly*[tiab] OR “plasminogen activator*”[tiab] OR alteplase[tiab] OR tenecteplase[tiab] OR reperfusion[tiab] OR “acute stroke care”[tiab] OR “acute stroke treatment*”[tiab]) ***(20,090)***

**#3** ("Health Services Administration"[Mesh] OR "Time-to-Treatment"[Mesh] OR “Time Factors”[Mesh]

OR "Regional Health Planning/organization and administration"[Mesh] OR "Delivery of Health Care, Integrated/economics"[Mesh] OR "Ambulatory Care Facilities/economics"[Mesh] OR "Ambulatory Care Facilities/organization and administration"[Mesh] OR “Emergency Medical Services”[Mesh] OR “Workflow”[Mesh] OR “health service*”[tiab] OR time*[tiab] OR “regional health plan*”[tiab] OR “health care plan*”[tiab] OR “delivery*”[tiab] OR ambula*[tiab] OR “organizational model*”[tiab] OR “geographic model*”[tiab] OR reconfigur*[tiab] OR (drip[tiab] *AND* ship[tiab]) OR mothership*[tiab] OR (trip[tiab] *AND* treat[tiab]) OR “mobile stroke unit*”[tiab] OR MSU [tiab] OR “mobile unit*”[tiab] OR “mobile CT unit*”[tiab] OR (drip[tiab] *AND* drive [tiab]) OR (drive[tiab] *AND* retrieve [tiab]) OR transfer*[tiab] OR transport*[tiab] OR dispatch*[tiab] OR “primary stroke cent*”[tiab] OR “comprehensive stroke cent*”[tiab] OR “EVT cent*”[tiab] OR “EVT-capable cent*”[tiab] OR delay*[tiab] OR prehospital[tiab] OR “pre-hospital”[tiab] OR “stroke alert”[tiab] OR emergenc*[tiab] OR triage[tiab] OR “hub-and-spoke model”[tiab] OR “hub cent*”[tiab] OR “spoke cent*”[tiab] OR telemedicine[tiab] OR train*[tiab] OR educat*[tiab] OR “pre-notification”[tiab] OR prenotification[tiab] OR workflow*[tiab] OR “work flow*”[tiab]) ***(338,871)***

**#4** ("Cost-Benefit Analysis"[Mesh] OR “cost effectiveness”[tiab] OR “cost utilit*”[tiab] OR “cost benefit*”[tiab] OR “economic outcome*”[tiab] OR “economic evaluation*”[tiab] OR “economic impact*”[tiab] OR “health economic*”[tiab] OR “economic modelling”[tiab] OR “economic assessment*”[tiab] OR “Quality-Adjusted Life Years”[Mesh] OR “quality-adjusted life year*”[tiab] OR QALY* [tiab] OR ICER[tiab] OR “incremental cost effectiveness ratio”[tiab]) ***(6,102)***

**#1 AND #2 AND #3 AND #4 *(29)***

**EMBASE (access on January 9^th^ 2022)**

**#1** ‘cerebrovascular accident’/exp OR (‘stroke*’ OR ‘cerebrovascular accident*’ OR ‘CVA’ OR ‘CVAs’ OR ‘cerebrovascular event*’ OR ‘brain vascular accident*’ OR ‘brain infarc*’ OR ‘brain ischemi*’ OR ‘brain attack’ OR ‘brain accident*’ OR ‘cerebral infarc*’ OR ‘cerebral ischemi*’ OR ‘cerebral vessel occlusion*’ OR ‘large vessel occlusion*’ OR ‘intracranial ischemi*’ OR ‘intracranial infarction*’ OR ‘intracranial vessel occlusion*’ OR ‘brain vessel occlusion*’):ab,ti AND [2021-2022]/py AND [01-07-2021]/sd NOT [10-01-2022]/sd ***(27,050)***

**#2** ‘thrombectomy’/exp OR ‘fibrinolytic therapy’/exp OR ‘plasminogen activator’/exp OR ‘reperfusion’/exp OR (‘thrombectom*’ OR ‘EVT’ OR ‘endovascular’ OR ‘IVT’ OR ‘thrombo*’ OR ‘fibrinoly*’ OR ‘alteplase’ OR ‘tenecteplase’ OR ‘plasminogen activator*’ OR ‘reperfusion’ OR ‘acute stroke care’ OR ‘acute stroke treatment*’):ab,ti AND [2021-2022]/py AND [01-07-2021]/sd NOT [10-01-2022]/sd ***(31,814)***

**#3** ‘health service’/exp OR ‘time to treatment’/exp OR ‘time factor’/exp OR ‘health care planning’/exp OR ‘health care delivery’/exp OR ‘workflow’/exp OR

(‘health service*’ OR ‘regional health plan*’ OR ‘health care plan*’ OR ‘delivery*’ OR ‘ambula*’ OR ‘organizational model*’ OR ‘geographic model*’ OR (‘drip’ AND ‘ship’) OR ‘mothership*’ OR (‘trip’ AND ‘treat’) OR ‘mobile stroke unit*’ OR ‘MSU’ OR ‘mobile unit*’ OR ‘mobile CT unit’ OR (‘drip’ *AND* ‘drive’) OR (‘drive’ *AND* ‘retrieve’) OR ‘transfer*’ OR ‘transport*’ OR ‘dispatch*’ OR ‘primary stroke cent*’ OR ‘comprehensive stroke cent*’ OR ‘EVT cent*’ OR ‘EVT-capable cent*’ OR ‘time*’ OR ‘delay*’ OR ‘prehospital’ OR ‘pre-hospital’ OR ‘stroke alert’ OR ‘emergenc*’ OR ‘triage’ OR ‘hub-and-spoke model’ OR ‘hub cent*’ OR ‘spoke cent*’ OR ‘telemedicine’ OR ‘train*’ OR ‘educat*’ OR ‘pre-notification’ OR ‘prenotification’ OR ‘workflow*’ OR ‘work flow*’):ab,ti AND [2021-2022]/py AND [01-07-2021]/sd NOT [10-01-2022]/sd ***(543,822)***

**#4** ‘cost benefit analysis’/exp OR ‘cost effectiveness analysis’/exp OR ‘cost utility analysis’/exp OR ‘quality adjusted life year’/exp OR (‘cost effectiveness’ OR ‘cost utilit*’ OR ‘cost benefit*’ OR ‘economic outcome*’ OR ‘economic evaluation*’ OR ‘economic impact*’ OR ‘health economic*’ OR ‘economic modelling’ OR ‘economic assessment*’ OR ‘quality adjusted life year*’ OR ‘QALY*’ OR ‘ICER’ OR ‘incremental cost effectiveness ratio’):ab,ti AND [2021-2022]/py AND [01-07-2021]/sd NOT [10-01-2022]/sd ***(9,700)***

**# 1 AND #2 AND #3 AND #4 *(50)***

**Web of Science (access on January 9^th^ 2022)**

Publication date from 01-07-2021 to 09-01-2022

**#1** TS=(stroke OR ‘cerebrovascular accident’ OR CVA OR CVAs OR ‘cerebrovascular event’ OR ‘brain vascular accident’ OR ‘brain infarc*’ OR ‘brain ischemi*’ OR ‘brain attack’ OR ‘brain accident*’ OR ‘cerebral infarc*’ OR ‘cerebral ischemi*’ OR ‘cerebral vessel occlusion’ OR ‘large vessel occlusion’ OR ‘intracranial ischemi*’ OR ‘intracranial infarction’ OR ‘intracranial vessel occlusion’ OR ‘brain vessel occlusion’) ***(20,641)***

**#2** TS=(‘thrombectom*’ OR ‘EVT’ OR ‘endovascular’ OR ‘IVT’ OR ‘thrombo*’ OR ‘fibrinoly*’ OR ‘alteplase’ OR ‘tenecteplase’ OR ‘plasminogen activator*’ OR ‘reperfusion’ OR ‘acute stroke care’ OR ‘acute stroke treatment*’) ***(37,676)***

**#3** TS=(‘health service*’ OR ‘regional health plan*’ OR ‘health care plan*’ OR ‘delivery*’ OR ‘ambula*’ OR ‘organizational model*’ OR ‘geographic model*’ OR (‘drip’ AND ‘ship’) OR ‘mothership*’ OR (‘trip’ AND ‘treat’) OR ‘mobile stroke unit*’ OR ‘MSU’ OR ‘mobile unit*’ OR ‘mobile CT unit’ OR (‘drip’ *AND* ‘drive’) OR (‘drive’ *AND* ‘retrieve’) OR ‘transfer*’ OR ‘transport*’ OR ‘dispatch*’ OR ‘primary stroke cent*’ OR ‘comprehensive stroke cent*’ OR ‘EVT cent*’ OR ‘EVT-capable cent*’ OR ‘time*’ OR ‘delay*’ OR ‘prehospital’ OR ‘pre-hospital’ OR ‘stroke alert’ OR ‘emergenc*’ OR ‘triage’ OR ‘hub-and-spoke model’ OR ‘hub cent*’ OR ‘spoke cent*’ OR ‘telemedicine’ OR ‘train*’ OR ‘educat*’ OR ‘pre-notification’ OR ‘prenotification’ OR ‘workflow*’ OR ‘work flow*’) ***(554,148)***

**#4** TS=(‘cost effectiveness’ OR ‘cost utilit*’ OR ‘cost benefit*’ OR ‘economic outcome*’ OR ‘economic evaluation*’ OR ‘economic impact*’ OR ‘health economic*’ OR ‘economic modelling’ OR ‘economic assessment*’ OR ‘quality adjusted life year*’ OR ‘QALY*’ OR ‘ICER’ OR ‘incremental cost effectiveness ratio’) ***(49,481)***

**#1 AND #2 AND #3 AND #4 (96)**

**Supplementary Table S1** Sensitivity analysis of included studies

| **Study** | **Intervention vs. comparator** | **Sensitivity analysis or scenario analysis** | **Results** |
| --- | --- | --- | --- |
| Ajmi (2021) [1] | Quality improvement (QI) project vs. no QI project | Scenario analysis:  Scenario 1: exclude unpaid time within working hours and outside of working hours  Scenario 2: exclude unpaid time within working hours and include unpaid time outside of working hours | Scenario 1: $13/minute door-to-needle time reduction and $4,679/ death averted  Scenario 2: $14/minute door-to-needle time reduction and $4,961/ death averted |
| Tan (2021) [2] | Telemedicine between hub and spoke centers vs. no telemedicine | 1 –way sensitivity, probabilistic sensitivity analysis  Scenario analysis with 5-year time horizon 5 years | 1-way sensitivity analysis: the probability of receiving tPA in a telestroke network, and stroke hospital costs-transferred have high impact on ICER  Probabilistic sensitivity analysis: telestroke is still dominant ($599 cost saving, 0.0817 QALY)  Scenario analysis: telestroke still is dominant ($2550 cost saving, 0.0299 QALY) |
| Coughlan (2021) [3] | Helicopter EMS vs. ground EMS | Probabilistic sensitivity  Scenario analysis: alternative time-to-treatment, 1-way sensitivity of alternative time horizon (1-20 year), eligible for thrombectomy, late presenters (after 6 hours) | Helicopter EMS had 62.9% probability of being cost-effective at £30,000 per QALY threshold.  Lower time horizon, higher ICER  Lower reduction time, higher ICER. Helicopter is only cost-effective at reduction of at least 60 min.  Higher eligibility for thrombectomy, lower ICER.  Helicopter EMS is dominated by ground EMS for late presenters. |
| Kim (2021) [4] | Mobile stroke unit (MSU) vs. standard ambulance and hospital stroke care pathway | 1-way sensitivity, probabilistic and multivariable sensitivity analyses  Scenario analysis: operation from 8 a.m. to 8 p.m., nurse-led telemedicine model and neurologist provided by telemedicine. | MSU has 95% of cost between $AU21,142 and $AU47,517 per DALY avoided  ICER was $AU 31,892 in case operation until 8 p.m., $AU 26,637 per DALY avoided in case nurse consultant, $AU 27,137 per DALY avoided in case nurse practitioner. In the probabilistic analysis of the nurse-led telemedicine model for MSU, 95% of MSU of cost being between $AU19,003 to $AU44,255 per DALY avoided. |
| Morii (2021) [5] | Drive and retrieve system vs. current practice | 1-way sensitivity analysis on specialist personnel and transportation cost | Hokumo area: ICER ranged from about $50,000 to $250,000.  Kamikawachubu area: ICER ranged from about -$10,000 to $50,000.  Nishiiburi area: ICER ranged from about $370,000 to $1,250,000. |
| Bayer (2020) [6] | A public information campaign to raise awareness of stroke symptoms and urgency  (combined with other interventions) vs. current practice | 1-way sensitivity analysis and probabilistic sensitivity analysis (Parameters were varied by  ±10%, assuming a random uniform distribution) | Sensitivity analyses showed that everything combined strategy was cost-effective when compared to current practice (Net monetary benefit is $SG324,113,685) |
| McMeekin (2019) [7] | 30 thrombectomy stroke centers vs. 24 thrombectomy stroke centers | 1-way sensitivity analysis and probabilistic sensitivity analysis | Using a common tariff for secondary transfer reduced net benefit by £20,400 and remained QALY outcomes.  Increasing eligible patients for EVT increased QALYs and saved further healthcare costs and via versa.  Reducing the mean age of patients increased QALYs and saved more health costs and via versa.  30 thrombectomy stroke centers has 100% of being cost-effective when compared to 24 centers at the threshold of £30,000. |
| Stevens (2019) [8] | **1/** enhanced educational material (EE)  **2/** interactive intervention (II)  Vs. standard care (SC) | 1-way sensitivity analysis and probabilistic sensitivity analysis | The ICER was most sensitive to the costs associated with informal caretakers and tPA, variation in the time horizon, and the benefits of tPA.  There was a 94.9% probability that II would be cost-effective versus EE and SC, a 5.1% probability that SC would be more cost-effective than II and EE at a threshold of $100,000/QALY. |
| Whetten (2018)[9] | Access to critical cerebral emergency support services (ACCESS) vs. standard care | 1-way sensitivity analysis and probabilistic sensitivity analysis | The ICER was sensitive to the cost of transfer, the probability of having the scan in less than 3 hours, and the probability of receiving tPA.  Probabilistic sensitivity analysis showed that mean cost saving per patient was $4,197 ($3,952-$4,438) and QALY gained per patient was 0.18 (0.14 – 0.22). |
| Yan (2018) [10] | Combine different transportation:  -Mothership by ground/ flight  -drip-and-ship by ground/ flight to minimum time to alteplase  -drip-and-ship by ground/flight to minimum time to EVT  Vs. mothership by ground | 1-way sensitivity analysis  Scenario analysis: % changing of affected population by switching optimal outcomes or optimal system value | The ICER was sensitive to the accuracy of diagnostic examination at the primary stroke center.  ICERs of optimal system value dominated mothership by ground.  ICERs of optimal clinical outcomes ranged from $16,463 to $55,213 when compared to mothership by ground. |
| Lahr (2017) [11] | **1/** Improving stroke care at 9 community hospitals to stroke centers;  **2/** 4 stroke centers and 5 hospitals without thrombolysis  **3/** 2 stroke centers and 7 hospitals without thrombolysis  Vs. current situation (9 community hospitals with thrombolysis) | 1-way sensitivity analysis (travel time) | Centralization of thrombolysis to 4 stroke centers: a decrease of 25% in travel time increased 0.4% of patients treated with thrombolysis.  Centralization of thrombolysis to 2 stroke centers: a decrease of 25% in travel time increased 0.5% of patients treated with thrombolysis.  Not reported sensitivity analysis of ICERs |
| Goff-Pronost (2017) [12] | 8 SUs without teleconsultation vs. 3 SUs and teleconsultation with emergency services in 5 hospitals | Sensitivity analyses were conducted to explore the impact of some variables (the rate of thrombolytic therapy, costs, rate of transfer to the stroke unit, rate of return home). | Not reported sensitivity analysis of ICERs |
| Espinoza  (2017) [13] | Standard stroke care supplemented with in-ambulance telemedicine vs. standard care | 1-way sensitivity analysis, 2-way sensitivity analysis (time reduction and costs), and probabilistic sensitivity analysis | 1-way sensitivity analysis at 12 min time gain showed that parameters related to outcome of ischemic stroke had the largest impact on costs and QALYs.  2-way sensitivity analysis: if implementation costs were more than $500,000, in-ambulance telemedicine was cost-effective after 19 min and dominant after 39 min. If implementation costs were less than $70,000, in-ambulance telemedicine was cost-effective after 3 min and dominant after 7 min.  Probabilistic sensitivity analysis: 90% of simulations were cost-effective at the threshold of $47,747, starting from 15 min time gain. |
| Torabi (2016) [14] | **1/** Partial telemedicine + stroke physician location at home  **2/** All telemedicine + stroke physician location at home  **3/** No telemedicine + stroke physician location at centre  **4/** Partial telemedicine + physician location at centre  **5/** All telemedicine + physician location at centre  Vs. No telemedicine+ stroke physician location at home | Sensitivity analyses were conducted on some variables (workup duration, longer telemedicine setup duration, ED triage duration, and traffic conditions). | % treated within 3 hours did not change much (1%) with different workup, telemedicine setup, triage, and traffic conditions (ambulance travels 12% faster with light and siren on or traffic moves 10% slower during rush hour).  Not reported sensitivity analysis in ICERs. |
| Gyrd-Hansen (2015) [15] | Stroke emergency mobile vs. normal EMS | Sensitivity analysis was undertaken by using a log-normal distribution of number needed to treat. | Sensitivity analysis estimated mean ICER of €31,814 (95% CI €15,909–€56,748). |
| Penaloza-Ramos (2014) [16] | **1/** Divert GP calls to ambulance service  **2/** Reduce time to call emergency service (series of educational interventions)  **6/** Immediate CT scan (CT scanner moved closer to the emergency department ward)  Vs. current practice | 1-way sensitivity analyses (costs) | The ranking of cost-effectiveness of the strategies did not alter under 1-way sensitivity analysis of long-term acute stroke costs, CT scan costs, and cost of thrombolysis drugs. |
| Dietrich (2014) [17] | Mobile stroke unit vs. normal EMS | Scenario analysis (different staffing, operating distances, population density) | Benefit-cost ratios increased as the staff size was reduced, up to a benefit-cost ratio of 3.71 in scenario 5 (1 paramedic, 1 radiology technician).  The optimal distances for highest benefit-cost ratios varied according to the staffing scenarios.  At an operating distance of 30km, the benefit-cost ratios increased with population density for all staffing scenarios. |
| McMeekin (2013) [18] | Central provision (2 regional neuroscience centers) vs. local ( 10 acute SUs) | 1-way sensitivity analysis (cost of ambulance per mile, % early presenting patients with moderate-to severe stroke, age of patients) , and probabilistic sensitivity analysis | Net benefit at £25,000 for QALY in sensitivity analysis:   - Use of ambulance Tariff: £20,000 - 1% increase/descrease in large artery occlusion: £93,000/ -£72,000 - Mean age -5 years/+5 years: £1,023,000/-£934,000   100% of simulations were cost-effective at the threshold of £25,000. |
| Demaerschalk (2013) [19] | Hub-and-spoke telestroke network vs. no network between hub and spokes | 1-way sensitivity analysis (recurrent stroke rate, transition probability of disability or death after recurrent stroke, utility, costs, network characteristics) and 2-way sensitivity analysis (spoke-to-hub transfer rate and endovascular rate among transferred patients) | 1-way sensitivity analyses showed that the results were robust ( a telestroke network being the dominant strategy in all scenarios) except when the spoke-to-hub transfer rate was varied.  2-way sensitivity analyses showed that the ICER was not sensitive to changes in the endovascular rate. |
| Switzer (2013) [20] | Hub-and-spoke telestroke network vs. no network between hub and spokes | 1-way sensitivity analysis (network characteristics, setup and maintenance costs of telestroke system, costs for treating patient, discharge dispositions with endovascular therapy) and 2-way sensitivity analysis (spoke-to-hub transfer rate and endovascular rate among transferred patients) | 1-way sensitivity analysis showed that the results were robust overall.  2-way sensitivity analysis showed that with 50% and 75% reductions in the endovascular rate, the network could achieve cost savings if the spoke-to-hub transfer rate was <62% and <43%, respectively. |

*EMS: emergency medical service, QALY: quality adjusted life year, ICER: incremental cost-effectiveness ratio, DALY: disability adjusted life year, tPA: tissue plasminogen activator, EVT: endovascular thrombectomy, CT: computed tomography*

**Supplementary Fig. S1** Quality assessment of included studies according to each item of the CHEERS statement

**Supplementary Table S2** PRISMA checklist

| **Section and Topic** | **Item #** | **Checklist item** | **Location where item is reported** |
| --- | --- | --- | --- |
| **TITLE** | | |  |
| Title | 1 | Identify the report as a systematic review. | Title page |
| **ABSTRACT** | | |  |
| Abstract | 2 | See the PRISMA 2020 for Abstracts checklist. | Title page |
| **INTRODUCTION** | | |  |
| Rationale | 3 | Describe the rationale for the review in the context of existing knowledge. | Page 3 |
| Objectives | 4 | Provide an explicit statement of the objective(s) or question(s) the review addresses. | Page 3 |
| **METHODS** | | |  |
| Eligibility criteria | 5 | Specify the inclusion and exclusion criteria for the review and how studies were grouped for the syntheses. | Page 3 |
| Information sources | 6 | Specify all databases, registers, websites, organisations, reference lists and other sources searched or consulted to identify studies. Specify the date when each source was last searched or consulted. | Page 3 |
| Search strategy | 7 | Present the full search strategies for all databases, registers and websites, including any filters and limits used. | Page 4, appendix 1 |
| Selection process | 8 | Specify the methods used to decide whether a study met the inclusion criteria of the review, including how many reviewers screened each record and each report retrieved, whether they worked independently, and if applicable, details of automation tools used in the process. | Page 4 |
| Data collection process | 9 | Specify the methods used to collect data from reports, including how many reviewers collected data from each report, whether they worked independently, any processes for obtaining or confirming data from study investigators, and if applicable, details of automation tools used in the process. | Page 4,5 |
| Data items | 10a | List and define all outcomes for which data were sought. Specify whether all results that were compatible with each outcome domain in each study were sought (e.g. for all measures, time points, analyses), and if not, the methods used to decide which results to collect. | Page 4,5 |
|  | 10b | List and define all other variables for which data were sought (e.g. participant and intervention characteristics, funding sources). Describe any assumptions made about any missing or unclear information. | Page 4,5 |
| Study risk of bias assessment | 11 | Specify the methods used to assess risk of bias in the included studies, including details of the tool(s) used, how many reviewers assessed each study and whether they worked independently, and if applicable, details of automation tools used in the process. | Page 5 |
| Effect measures | 12 | Specify for each outcome the effect measure(s) (e.g. risk ratio, mean difference) used in the synthesis or presentation of results. | Page 4,5 |
| Synthesis methods | 13a | Describe the processes used to decide which studies were eligible for each synthesis (e.g. tabulating the study intervention characteristics and comparing against the planned groups for each synthesis (item #5)). | Page 4,5 |
|  | 13b | Describe any methods required to prepare the data for presentation or synthesis, such as handling of missing summary statistics, or data conversions. | Page 4,5 |
|  | 13c | Describe any methods used to tabulate or visually display results of individual studies and syntheses. | Page 4,5 |
|  | 13d | Describe any methods used to synthesize results and provide a rationale for the choice(s). If meta-analysis was performed, describe the model(s), method(s) to identify the presence and extent of statistical heterogeneity, and software package(s) used. | NA |
|  | 13e | Describe any methods used to explore possible causes of heterogeneity among study results (e.g. subgroup analysis, meta-regression). | NA |
|  | 13f | Describe any sensitivity analyses conducted to assess robustness of the synthesized results. | NA |
| Reporting bias assessment | 14 | Describe any methods used to assess risk of bias due to missing results in a synthesis (arising from reporting biases). | NA |
| Certainty assessment | 15 | Describe any methods used to assess certainty (or confidence) in the body of evidence for an outcome. | NA |
| **RESULTS** | | |  |
| Study selection | 16a | Describe the results of the search and selection process, from the number of records identified in the search to the number of studies included in the review, ideally using a flow diagram. | Page 5 |
|  | 16b | Cite studies that might appear to meet the inclusion criteria, but which were excluded, and explain why they were excluded. | NA |
| Study characteristics | 17 | Cite each included study and present its characteristics. | Page 5,6, table 1 |
| Risk of bias in studies | 18 | Present assessments of risk of bias for each included study. | Page 7, table 2, supplementary Fig. S1 |
| Results of individual studies | 19 | For all outcomes, present, for each study: (a) summary statistics for each group (where appropriate) and (b) an effect estimate and its precision (e.g. confidence/credible interval), ideally using structured tables or plots. | Page 8,9, table 3 |
| Results of syntheses | 20a | For each synthesis, briefly summarise the characteristics and risk of bias among contributing studies. | Page 8,9, table 3 |
|  | 20b | Present results of all statistical syntheses conducted. If meta-analysis was done, present for each the summary estimate and its precision (e.g. confidence/credible interval) and measures of statistical heterogeneity. If comparing groups, describe the direction of the effect. | NA |
|  | 20c | Present results of all investigations of possible causes of heterogeneity among study results. | NA |
|  | 20d | Present results of all sensitivity analyses conducted to assess the robustness of the synthesized results. | NA |
| Reporting biases | 21 | Present assessments of risk of bias due to missing results (arising from reporting biases) for each synthesis assessed. | NA |
| Certainty of evidence | 22 | Present assessments of certainty (or confidence) in the body of evidence for each outcome assessed. | NA |
| **DISCUSSION** | | |  |
| Discussion | 23a | Provide a general interpretation of the results in the context of other evidence. | Page 9,10 |
|  | 23b | Discuss any limitations of the evidence included in the review. | Page 11,12 |
|  | 23c | Discuss any limitations of the review processes used. | Page 11 |
|  | 23d | Discuss implications of the results for practice, policy, and future research. | Page 10,11,12 |
| **OTHER INFORMATION** | | |  |
| Registration and protocol | 24a | Provide registration information for the review, including register name and registration number, or state that the review was not registered. | Page 13 |
|  | 24b | Indicate where the review protocol can be accessed, or state that a protocol was not prepared. | Page 13 |
|  | 24c | Describe and explain any amendments to information provided at registration or in the protocol. | NA |
| Support | 25 | Describe sources of financial or non-financial support for the review, and the role of the funders or sponsors in the review. | Page 13 |
| Competing interests | 26 | Declare any competing interests of review authors. | Page 13 |
| Availability of data, code and other materials | 27 | Report which of the following are publicly available and where they can be found: template data collection forms; data extracted from included studies; data used for all analyses; analytic code; any other materials used in the review. | Page 13 |

*From:*  Page MJ, McKenzie JE, Bossuyt PM, Boutron I, Hoffmann TC, Mulrow CD, et al. The PRISMA 2020 statement: an updated guideline for reporting systematic reviews. BMJ 2021;372:n71. doi: 10.1136/bmj.n71

**Supplementary Table S3** PRISMA 2020 for Abstract Checklist

| **Section and Topic** | **Item #** | **Checklist item** | **Reported (Yes/No)** |
| --- | --- | --- | --- |
| **TITLE** | | |  |
| Title | 1 | Identify the report as a systematic review. | Yes |
| **BACKGROUND** | | |  |
| Objectives | 2 | Provide an explicit statement of the main objective(s) or question(s) the review addresses. | Yes |
| **METHODS** | | |  |
| Eligibility criteria | 3 | Specify the inclusion and exclusion criteria for the review. | Yes |
| Information sources | 4 | Specify the information sources (e.g. databases, registers) used to identify studies and the date when each was last searched. | Yes |
| Risk of bias | 5 | Specify the methods used to assess risk of bias in the included studies. | Yes |
| Synthesis of results | 6 | Specify the methods used to present and synthesise results. | No |
| **RESULTS** | | |  |
| Included studies | 7 | Give the total number of included studies and participants and summarise relevant characteristics of studies. | Yes |
| Synthesis of results | 8 | Present results for main outcomes, preferably indicating the number of included studies and participants for each. If meta-analysis was done, report the summary estimate and confidence/credible interval. If comparing groups, indicate the direction of the effect (i.e. which group is favoured). | Yes |
| **DISCUSSION** | | |  |
| Limitations of evidence | 9 | Provide a brief summary of the limitations of the evidence included in the review (e.g. study risk of bias, inconsistency and imprecision). | No |
| Interpretation | 10 | Provide a general interpretation of the results and important implications. | Yes |
| **OTHER** | | |  |
| Funding | 11 | Specify the primary source of funding for the review. | No |
| Registration | 12 | Provide the register name and registration number. | No |

**References**

1. Ajmi SC, Kurz MW, Ersdal H, Lindner T, Goyal M, Issenberg SB, Vossius C: **Cost-effectiveness of a quality improvement project, including simulation-based training, on reducing door-to-needle times in stroke thrombolysis**. *BMJ quality & safety* 2021.

2. Tan E, Gao L, Tran HN, Cadilhac D, Bladin C, Moodie M: **Telestroke for acute ischaemic stroke: A systematic review of economic evaluations and a de novo cost-utility analysis for a middle income country**. *Journal of telemedicine and telecare* 2021:1357633x211032407.

3. Coughlan D, McMeekin P, Flynn D, Ford GA, Lumley H, Burgess D, Balami J, Mawson A, Craig D, Rice S *et al*: **Secondary transfer of emergency stroke patients eligible for mechanical thrombectomy by air in rural England: economic evaluation and considerations**. *Emergency medicine journal : EMJ* 2021, **38**(1):33-39.

4. Kim J, Easton D, Zhao H, Coote S, Sookram G, Smith K, Stephenson M, Bernard S, M WP, Yan B *et al*: **Economic evaluation of the Melbourne Mobile Stroke Unit**. *International journal of stroke : official journal of the International Stroke Society* 2021, **16**(4):466-475.

5. Morii Y, Osanai T, Fujiwara K, Tanikawa T, Houkin K, Gu S, Ogasawara K: **Analyzing Cost-Effectiveness of Allocating Neurointerventionist for Drive and Retrieve System for Patients with Acute Ischemic Stroke**. *Journal of stroke and cerebrovascular diseases : the official journal of National Stroke Association* 2021, **30**(8):105843.

6. Bayer S, Eom K, Sivapragasam N, Silva DAD, Choon G, Koh H, Tan KB, Ansah JP, Matchar DB: **Estimating costs and benefits of stroke management: A population-based simulation model**. *Journal of the Operational Research Society* 2020, **72**(9):2122-2134.

7. McMeekin P, Flynn D, Allen M, Coughlan D, Ford GA, Lumley H, Balami JS, James MA, Stein K, Burgess D *et al*: **Estimating the effectiveness and cost-effectiveness of establishing additional endovascular Thrombectomy stroke Centres in England: a discrete event simulation**. *BMC health services research* 2019, **19**(1):821.

8. Stevens ER, Roberts E, Kuczynski HC, Boden-Albala B: **Stroke Warning Information and Faster Treatment (SWIFT): Cost-Effectiveness of a Stroke Preparedness Intervention**. *Value in health : the journal of the International Society for Pharmacoeconomics and Outcomes Research* 2019, **22**(11):1240-1247.

9. Whetten J, van der Goes DN, Tran H, Moffett M, Semper C, Yonas H: **Cost-effectiveness of Access to Critical Cerebral Emergency Support Services (ACCESS): a neuro-emergent telemedicine consultation program**. *Journal of medical economics* 2018, **21**(4):398-405.

10. Yan C, Zheng Y, Hill MD, Mann B, Jeerakathil T, Kamal N, Amlani S, Chuck AW: **Health Technology Optimization Analysis: Conceptual Approach and Illustrative Application**. *MDM policy & practice* 2018, **3**(1):2381468318774804.

11. Lahr MM, van der Zee DJ, Luijckx GJ, Vroomen PC, Buskens E: **Centralising and optimising decentralised stroke care systems: a simulation study on short-term costs and effects**. *BMC medical research methodology* 2017, **17**(1):5.

12. Pronost M, Benjamin B, Gantzer S, Moulin T: **Medico-economic modelling of stroke care with telemedicine. An experience in Franche-Comté**. *European Research in Telemedicine / La Recherche Européenne en Télémédecine* 2017, **6**.

13. Valenzuela Espinoza A, Devos S, van Hooff RJ, Fobelets M, Dupont A, Moens M, Hubloue I, Lauwaert D, Cornu P, Brouns R *et al*: **Time Gain Needed for In-Ambulance Telemedicine: Cost-Utility Model**. *JMIR mHealth and uHealth* 2017, **5**(11):e175.

14. Torabi E, Froehle CM, Lindsell CJ, Moomaw CJ, Kanter D, Kleindorfer D, Adeoye O: **Monte Carlo Simulation Modeling of a Regional Stroke Team's Use of Telemedicine**. *Academic emergency medicine : official journal of the Society for Academic Emergency Medicine* 2016, **23**(1):55-62.

15. Gyrd-Hansen D, Olsen KR, Bollweg K, Kronborg C, Ebinger M, Audebert HJ: **Cost-effectiveness estimate of prehospital thrombolysis: results of the PHANTOM-S study**. *Neurology* 2015, **84**(11):1090-1097.

16. Penaloza-Ramos MC, Sheppard JP, Jowett S, Barton P, Mant J, Quinn T, Mellor RM, Sims D, Sandler D, McManus RJ: **Cost-effectiveness of optimizing acute stroke care services for thrombolysis**. *Stroke* 2014, **45**(2):553-562.

17. Dietrich M, Walter S, Ragoschke-Schumm A, Helwig S, Levine S, Balucani C, Lesmeister M, Haass A, Liu Y, Lossius HM *et al*: **Is prehospital treatment of acute stroke too expensive? An economic evaluation based on the first trial**. *Cerebrovascular diseases (Basel, Switzerland)* 2014, **38**(6):457-463.

18. McMeekin P, Gray J, Ford GA, Rodgers H, Price CI: **Modelling the efficiency of local versus central provision of intravenous thrombolysis after acute ischemic stroke**. *Stroke* 2013, **44**(11):3114-3119.

19. Demaerschalk BM, Switzer JA, Xie J, Fan L, Villa KF, Wu EQ: **Cost utility of hub-and-spoke telestroke networks from societal perspective**. *The American journal of managed care* 2013, **19**(12):976-985.

20. Switzer JA, Demaerschalk BM, Xie J, Fan L, Villa KF, Wu EQ: **Cost-effectiveness of hub-and-spoke telestroke networks for the management of acute ischemic stroke from the hospitals' perspectives**. *Circulation Cardiovascular quality and outcomes* 2013, **6**(1):18-26.
